# The influence of elastic ankle exoskeletons on lower limb mechanical energetics during unexpected perturbations

**DOI:** 10.1098/rsos.221133

**Published:** 2023-02-01

**Authors:** James L. Williamson, Glen A. Lichtwark, Gregory S. Sawicki, Taylor J. M. Dick

**Affiliations:** ^1^ School of Biomedical Sciences, University of Queensland, St Lucia, Queensland 4072, Australia; ^2^ School of Human Movement and Nutrition Sciences, University of Queensland, St Lucia, Queensland 4072, Australia; ^3^ George W. Woodruff School of Mechanical Engineering and School of Biological Sciences, Georgia Institute of Technology, Atlanta, GA 30332, USA

**Keywords:** balance, biomechanics, inverse dynamics, joint work, locomotion, stability

## Abstract

Passive elastic ankle exoskeletons have been used to augment locomotor performance during walking, running and hopping. In this study, we aimed to determine how these passive devices influence lower limb joint and whole-body mechanical energetics to maintain stable upright hopping during rapid, unexpected perturbations. We recorded lower limb kinematics and kinetics while participants hopped with exoskeleton assistance (0, 76 and 91 Nm rad^−1^) on elevated platforms (15 and 20 cm) which were rapidly removed at an unknown time. Given that springs cannot generate nor dissipate energy, we hypothesized that passive ankle exoskeletons would reduce stability during an unexpected perturbation. Our results demonstrate that passive exoskeletons lead to a brief period of instability during unexpected perturbations — characterized by increased hop height. However, users rapidly stabilize via a distal-to-proximal redistribution of joint work such that the knee performs an increased energy dissipation role and stability is regained within one hop cycle. Together, these results demonstrate that despite the inability of elastic exoskeletons to directly dissipate mechanical energy, humans can still effectively dissipate the additional energy of a perturbation, regain stability and recover from a rapid unexpected vertical perturbation to maintain upright hopping.

## 1. Introduction

The behaviour with which lower limbs store and return energy during steady gait has inspired the design of passive ankle exoskeletons to augment locomotor performance during walking [1], running [2] and hopping [3]. Passive ankle exoskeletons that place a spring in parallel with the ankle plantar flexors and Achilles tendon have been shown to reduce the net metabolic cost of walking by approximately 7%, at an optimum stiffness [1]. Although the energetic benefits, via off-loading muscle force, of lower limb exoskeletons during steady-state conditions (constant walking speed or hopping frequency) on level ground are well established [1,3], it remains unresolved how similar devices influence the biomechanics of movement under the more variable and unpredictable conditions that humans encounter during everyday locomotion. Spring-like passive elastic exoskeletons reduce forces, but can neither generate nor dissipate net energy, and thus may reduce stability when navigating variable terrain. To better understand this, we explored how passive ankle exoskeletons influence lower limb joint and whole-body mechanical energetics to maintain stable upright hopping during rapid, unexpected changes in the height of the ground.

Humans must generate and dissipate net energy to maintain steady movement in complex and unpredictable environments (i.e. holes, bumps and curbs). Perturbations to human locomotion have been elicited via a number of experimental paradigms including visible and camouflaged changes in substrate height during overground walking and running [4–6], unexpected bumps [7] or removal of ground support during walking [8,9], and unexpected support-surface translations during standing balance [10]. Recently, we investigated how humans adjust lower limb mechanics [11] and neuromuscular control [12] to negotiate rapid, unexpected vertical perturbations (5–20 cm) during hopping. Our results illustrated that humans recover from falling in a hole by increasing the energy absorbed predominantly in the distal ankle joint at small perturbation heights (5–10 cm) [11]. However, with increased perturbation height (20 cm), we observed a distal (ankle) to proximal (knee/hip) shift in energy absorption, with humans dissipating 1.4 times more energy across the whole lower limb than would be expected due to the change in ground height [11].

Given their elastic nature, spring-based passive exoskeletons can neither generate nor dissipate net energy [1,13]. However, a series of studies have highlighted that passive devices can influence an individual's capacity to generate or dissipate energy via modifying normal joint- and muscle-level behaviour. For example, during walking and hopping, passive ankle exoskeletons have been shown to alter joint kinematics and kinetics, muscle activity, and muscle fascicle dynamics [1,3,14,15]. During hopping with passive ankle exoskeleton assistance (91 Nm rad^−1^), the ankle becomes more plantarflexed while both the biological ankle moment and soleus muscle activity decrease [3,16]. To maintain ankle stiffness over a hop cycle, the antagonist tibialis anterior co-activates and increases activation to counteract the exoskeleton plantarflexion moment [3,16]. Together with musculoskeletal simulations [16], these studies demonstrate that passive ankle exoskeletons inherently influence an individual's capacity to generate and dissipate energy during hopping via disrupting joint- and muscle-level mechanical behaviour. However, these previous studies have predominantly focused on steady-state locomotor tasks, and thus we have a limited understanding of how similar devices influence the stability of movement and alter lower limb joint behaviour during unexpected, rapid perturbations.

In this study, we investigated how passive elastic ankle exoskeletons influence the mechanical energetics of the lower limb joints (ankle, knee and hip) and whole-body stability (as indicated by hop height) during unexpected vertical perturbations to human hopping. We used inverse dynamics to determine individual lower limb joint moments, work and power during two-legged hopping before and during rapid vertical perturbations with and without bilateral passive ankle exoskeleton assistance. We measured joint and whole-body level responses at three assistance levels (0, 76 and 91 Nm rad^−1^) and two changes in ground height (15 and 20 cm). Given that springs cannot generate nor dissipate energy, we hypothesized that passive ankle exoskeletons would reduce stability during an unexpected perturbation.

## 2. Methods

### 2.1. Exoskeleton design

**Figure 1.**
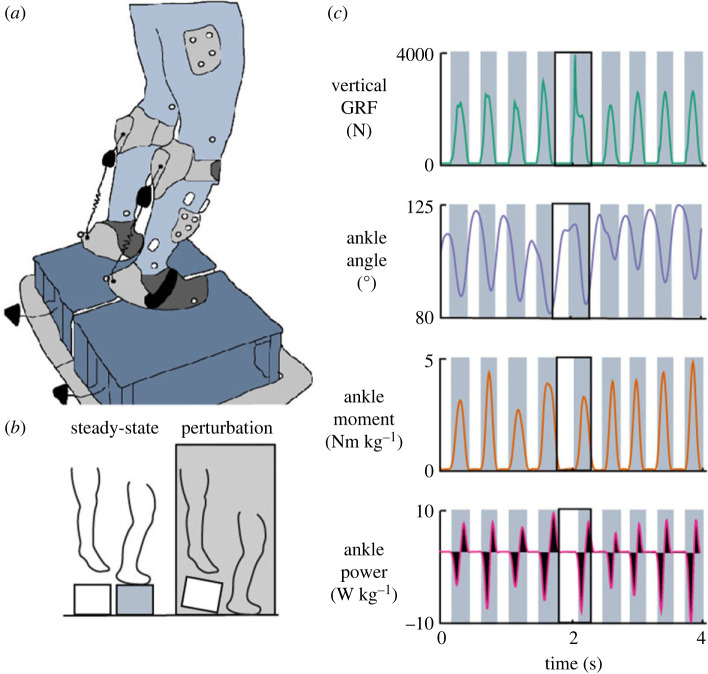
An illustration of the perturbation paradigm with passive ankle exoskeletons (*a*). Individuals hopped at their preferred frequency on a platform (15 or 20 cm) that was rapidly removed at an unknown time. (*b*) Data were analysed for the aerial and ground contact phases for both steady-state hopping (averaged for three hops) and the perturbation. (*c*) Lower limb kinematics were measured using motion capture and GRFs using two force plates. The shaded regions indicate periods of ground contact. The black outlined box represents the perturbation.

A soft passive ankle exoskeleton was designed for experimentation (figure 1*a*). This device was comprised of four components, a polycaprolactone shank attachment and foot cup, extension spring, and locking cam to set spring slack length on an individual basis. Two hoop-loop straps were embedded into the polycaprolactone matrix to secure the attachment to the lower leg and foam was attached to the shank to avoid discomfort. The moment arm of the foot attachment about the biological ankle joint averaged 101.1 ± 22.3 mm across participants. Exoskeletons were fit to each participant by the addition or removal of foam inserts at device-skin contact areas. Three assistance levels were selected for experimentation: a no assistance condition (0 Nm rad^−1^), 76 Nm rad^−1^ (121–365, RS Components) and 91 Nm rad^−1^ (751–944, RS Components). The 91 Nm rad^−1^ condition was selected based on the hopping-exoskeleton design by Farris *et al.* and represents approximately 40% of ankle stiffness during preferred hopping [17]. Similarly, the device slack-angle (the angle at which the extension spring begins to store elastic strain energy) was set to 127°, consistent with Farris *et al.* [17]. An ankle angle of 127° represents the average ankle angle at ground contact during steady-state hopping [17,18]. The torque provided by the exoskeleton during hopping was determined by multiplying an experimentally determined exoskeleton spring stiffness by dynamic spring displacements during hopping (as measured by reflective markers placed on the spring component of the device).

### 2.2. Experimental protocol

Eleven participants (4 male, 6 female, 24 ± 3 years, 67.5 ± 8.4 kg, 168.6 ± 10.7 cm, mean ± s.d.) provided written informed consent and completed an unexpected ‘falling-in-a-hole’ experimental paradigm, as previously described by Dick *et al*. [11]. Briefly, a vertical perturbation was elicited via the rapid removal of ground platforms (15 or 20 cm) during bilateral ankle-dominated hopping (figure 1). The platform was removed at an unknown time between the 10th and 20th hop.

Participants wore custom-made bilateral passive ankle exoskeletons during hopping. Participants hopped at their preferred frequency (2.17 ± 0.24 Hz) while wearing a safety harness but were not instructed to reach a target height and were not paced with a metronome. Conditions were randomized to test the effect of perturbation height (15 and 20 cm) and exoskeleton stiffness (0, 76 and 91 Nm rad^−1^). During each trial, kinematic and kinetic data were collected on the right leg (figure 1). All outcome measures were separated into steady-state hopping (averaged over three hops prior to the perturbation) and the perturbation (when the platform was removed). Hopping trials where the platforms were not completely removed from the force plates before ground contact were repeated.

### 2.3. Kinematics and kinetics

Lower limb kinematics were recorded via a 12-camera motion capture system (100 Hz, Flex3, Optitrack, USA). Individual reflective markers were placed bilaterally on bony landmarks of the lower limbs and pelvis. Custom three-dimensional printed rigid body clusters of four markers were secured to the left and right thigh and shank. This marker set is consistent with previous experimental protocols [19]. Participants hopped with each foot on a separate force plate such that one three-dimensional ground reaction force (GRF) vector could be attributed to each of the right and left lower limbs (2048 Hz, Bertec Corp., USA). Force plate data were collected via a data acquisition board (CED1401, CED Ltd., UK) and combined with motion capture data via a custom MATLAB script which used the BTK toolkit [20]. To account for the platform weight and position on the force plate during steady-state hopping, a custom MATLAB script was written that subtracted box weight from the vertical component of the GRF and projected the GRF to the intersection of the platform surface in three-dimensional space. Data were filtered with second-order low-pass Butterworth filters (motion capture data: 10 Hz; GRF: 25 Hz).

OpenSim was used to scale a musculoskeletal model using a static standing trial for each participant [21,22]. The subject-specific scaled model was used together with motion capture data in an inverse kinematics analysis to determine the time-varying joint angles for the ankle, knee and hip. These kinematics were combined with GRF's in an inverse dynamics analysis to determine the time-varying joint moments [21,22]. Biological ankle joint moments were calculated as the moment generated about the ankle subtracted by the estimated exoskeleton moment. Joint and exoskeleton moments were normalized to body mass.

### 2.4. Hop height as a proxy of stability

Hop heights were measured via the vertical displacement of a virtual marker placed midway between the left and right posterior superior iliac spines. For a given hop, the hop height was taken as the vertical displacement of this virtual marker from ground contact to the next aerial phase. A proxy of stability was defined as ±1 s.d. of the hop height during steady-state hopping in the no assistance condition (HHss, no exo). These criteria specify that if mean hop height was greater than HHss, no exo +1s.d. or less than HHss, no exo −1s.d., it was considered unsteady.

### 2.5. Mechanical energetics—joint work and power calculations

Mechanical work and powers for the right ankle, knee and hip joints were calculated. Briefly, ankle, knee and hip joint angular velocities were determined as the first derivative of ankle, knee and hip joint angle with respect to time, respectively. Instantaneous joint powers were determined as the product of joint angular velocities and joint moments. Positive joint power represents action to extend the joint and negative joint powers represent flexion. To determine joint work, the trapezium method was used to integrate joint power over periods of positive and negative work [11,23]. For each hop, negative and positive work at each individual joint were summed to determine net joint work. Joint work was normalized to body mass.

### 2.6. Mechanical energetics—centre of mass work calculations

Consistent with Smith *et al.,* periods of positive and negative centre of mass (COM) work were found by integrating time-varying COM power via the trapezium methods [24]. COM power was calculated as the dot product of the COM velocity and vertical GRF ($GRF_{v}$) (2.1). To determine the COM velocity, participant weight (body weight) was subtracted from the vertical component of the GRF, and the resultant force was divided by body mass and integrated via the trapezium method. The COM velocity integration constant was estimated to be the mean COM velocity. Finally, COM work was normalized to body mass.

2.1 $${\mathrm{COM}}_{\mathrm{power}}={\mathrm{GRF}}_{v}\;\int \frac{{\mathrm{GRF}}_{v}-\mathrm{body}\;\mathrm{weight}}{\mathrm{body}\;\mathrm{mass}}\;dt.$$

### 2.7. Statistics

Hop height descriptive statistics (mean, s.d.) before (steady-state), during (perturbation) and after (subsequent) the perturbation were used to assess the influence of passive ankle exoskeletons on whole-body stability via the application of the proxy of stability. Linear mixed-effects models were then used to assess the influence of exoskeleton stiffness (0, 76, 91 Nm rad^−1^) and condition (steady-state and perturbation) at the 15 and 20 cm perturbations on hopping metrics, joint kinematics and mechanics, and COM work. For steady-state hopping, measures for both the 15 and 20 cm conditions were combined. A within-participant design was used, including participant as a random factor using the lme.R function from the nlme package in R (v. 4.1.1, Vienna, Austria) [25,26]. The glht.R function from the multcomp package was used to perform Tukey *post hoc* tests. Differences were considered significant at *p* < 0.05.

## 3. Results

### 3.1. The effect of exoskeleton assistance on the perturbation response

**Figure 2.**
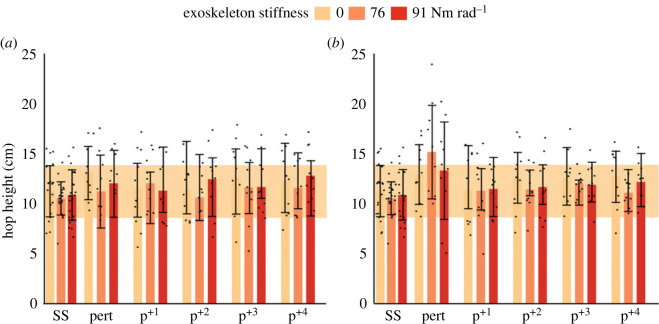
Hop heights during steady-state hopping (ss), the perturbation (pert) and four successive hops following the perturbation (p^+n^) for the 15 cm (*a*) and 20 cm (*b*) drop heights. Bar plots show mean hop height, with error bars representing the s.d. Bar plots are shaded according to the exoskeleton stiffness condition: 0 Nm rad^−1^ (light orange), 76 Nm rad^−1^ (orange) and 91 Nm rad^−1^ (red). The shaded box represents a proxy of stability, defined as ±1 s.d. of hop height during steady-state hopping with no assistance (0 Nm rad^−1^). Individual data points are shown as black circles.

The magnitude of the perturbation influenced stability with exoskeleton assistance (figure 2). When applying the proxy of stability, the 20 cm perturbation with assistance (76 Nm rad^−1^) was found to be unstable by comparison to the s.d. of the steady-state no assistance condition. However, stability was regained within one hop cycle (p^+1^) (figure 2*b*). Specifically, during the 20 cm perturbation, participants hopped 2.2 ± 0.67 cm higher with exoskeleton assistance (figure 2*b, p* = 0.003), whereby hop height was higher at the 76 Nm rad^−1^ condition, compared to no assistance (*p* = 0.002). During the 15 cm perturbation, hop height was lower with exoskeleton assistance (*p* = 0.014) while remaining within the proxy of stability, with a 1.8 ± 0.61 cm reduction in hop height at the 76 Nm rad^−1^ condition, compared to no assistance (figure 2, *p* = 0.007). There was no effect of exoskeleton assistance on hop height following the 15 or 20 cm perturbation (p^+1^ to p^+4^).

**Figure 3.**
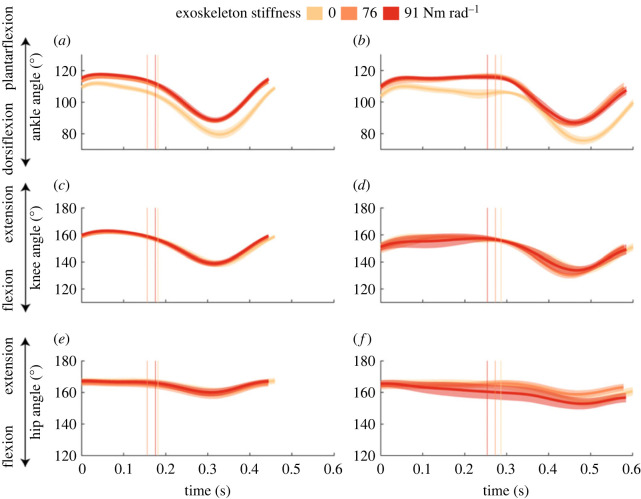
Group mean ankle (*a*,*b*), knee (*c*,*d*) and hip (*e*,*f*) joint angles during steady-state hopping (*a*,*c*,*e*) and the perturbation (*b*,*d*,*f*) for the 20 cm perturbation. The 0 Nm rad^−1^ (light orange), 76 Nm rad^−1^ (orange) and 91 Nm rad^−1^ (red) conditions are denoted by colour. s.e. is shown with the shaded area. Average ground contact times for each condition are shown with vertical lines, in a similar colour to the corresponding spring condition.

**Figure 4.**
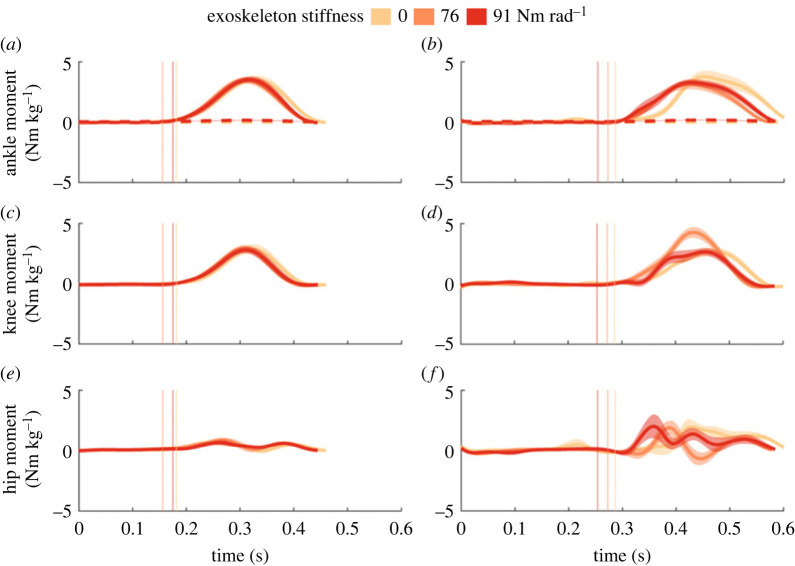
Group mean ankle (*a*,*b*), knee (*c*,*d*) and hip (*e*,*f*) joint moments during steady-state hopping (*a*,*c*,*e*) and the perturbation (*b*,*d*,*f*) for the 20 cm perturbation height. The 0 Nm rad^−1^ (light orange), 76 Nm rad^−1^ (orange) and 91 Nm rad^−1^ (red) conditions are denoted by colour. s.e. is shown with the shaded area. Average ground contact times are shown for each condition with vertical lines, in a similar colour to the corresponding spring condition. Exoskeleton moments for the 76 and 91 Nm rad^−1^ conditions provided on average 4.8% and 4.6% of the peak ankle moment during steady-state hopping. At the perturbation, exoskeleton moments for the 76 and 91 Nm rad^−1^ conditions supplied on average 3.8% and 4.7% of the peak ankle moment.

Lower limb kinematics and moments were influenced by exoskeleton assistance (electronic supplementary material, tables S1 and S2). During both steady-state hopping and the perturbation, exoskeleton assistance resulted in a generally more plantarflexed ankle with assistance (76 and 91 Nm rad^−1^) compared to no assistance (electronic supplementary material, table S1 and figure S1; figure 3). During the perturbation, knee and hip angles did not vary with exoskeleton assistance. Our results highlight changes in knee and hip joint moments with exoskeleton assistance (electronic supplementary material, table S2). During the 20 cm perturbation, peak knee extension moment was 1.42 ± 0.39 Nm kg^−1^ larger at the 76 Nm rad^−1^ condition, compared to no assistance (electronic supplementary material, table S2 and figure S2; figure 4, *p* = 0.004). Peak hip moments did not vary with exoskeleton assistance. Exoskeleton moments during steady-state hopping represented approximately 4.8% and 4.6% of peak biological ankle plantarflexion moment at the 76 and 91 Nm rad^−1^ conditions, respectively. Exoskeleton moments during the perturbation represented approximately 3.8% and 4.7% of the peak biological ankle plantarflexion moment at the 76 and 91 Nm rad^−1^ conditions, respectively.

**Figure 5.**
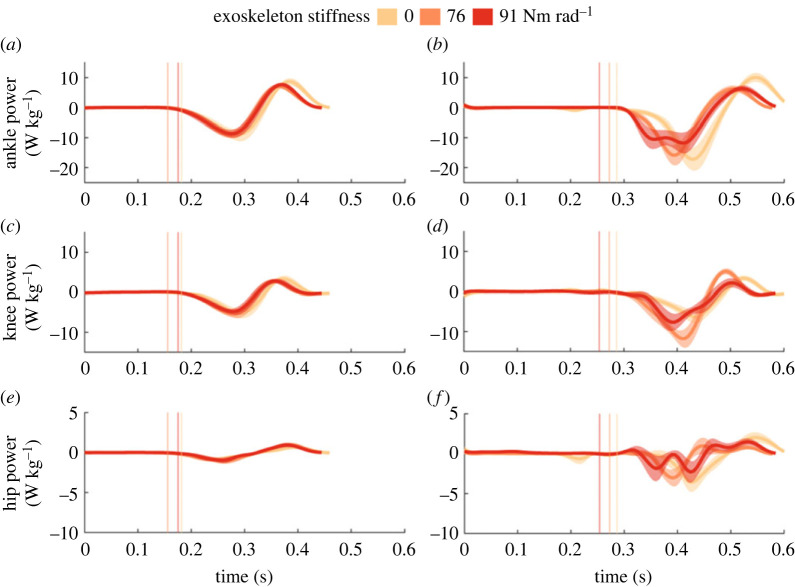
Group mean ankle (*a*,*b*), knee (*c*,*d*) and hip (*e*,*f*) joint power during steady-state hopping (*a*,*c*,*e*) and the perturbation (*b*,*d*,*f*) for the 20 cm perturbation. The 0 Nm rad^−1^ (light orange), 76 Nm rad^−1^ (orange) and 91 Nm rad^−1^ (red) conditions are denoted by colour. s.e. is shown with the shaded area. Average ground contact times for each condition are shown with vertical lines, in a similar colour to the corresponding spring condition.

**Figure 6.**
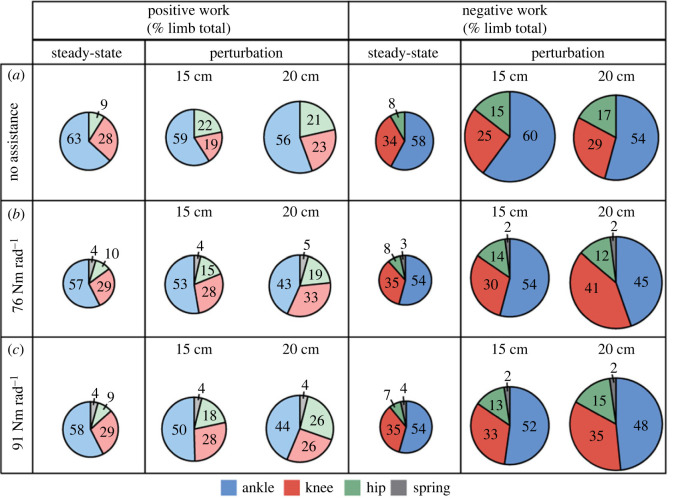
Relative contributions of the ankle, knee and hip joints to total lower limb work during steady-state hopping and the perturbation with no assistance 0 Nm rad^−1^ (*a*), 76 Nm rad^−1^ (*b*) and 91 Nm rad^−1^ (*c*) spring conditions. Pie charts display the percentage of total average positive work (left, lighter colours) and total average negative work (right, darker colours) contributed by the ankle (blue), knee (red), hip (green) and exoskeleton (grey). The diameter of each pie indicates the total positive or negative power relative to the steady-state no assistance (0 Nm rad^−1^) condition.

Exoskeleton assistance altered the magnitude and distribution of lower limb joint work during the perturbation (table 1). During the 15 cm perturbation, net ankle work increased (table 1, *p* = 0.043) with exoskeleton assistance, on average by 0.42 ± 0.15 J kg^−1^ at the 91 Nm rad^−1^ condition, compared to no assistance (*p =* 0.019). During the 20 cm perturbation, net knee work decreased on average by 0.36 ± 0.11 J kg^−1^ at the 76 Nm rad^−1^ condition, compared to no assistance (*p* = 0.005). This ankle, knee and hip work redistribution is temporally visualized as the area under each joint power curve in electronic supplementary material, figure S3 (15 cm perturbation) and figure 5 (20 cm perturbation). In total, the knee increased its contribution to total lower limb negative work from 29% (no assistance) to 42% at the 76 Nm rad^−1^ condition during the 20 cm perturbation (figure 6*b*). During the 15 cm perturbation, total lower limb work increased with exoskeleton assistance (*p* = 0.026) with on average 0.58 ± 0.19 J kg^−1^ more work done at the 91 Nm rad^−1^ condition (*p =* 0.007), compared to no assistance.

**Table 1.** Ankle, knee and hip joint work during steady-state hopping and the perturbation with and without exoskeleton assistance. Lower limb joint work for steady-state hopping and the perturbation (15 and 20 cm) at 0, 76 and 91 Nm rad^−1^ exoskeleton assistance. Values are reported as mean ± s.d.

	perturbation height	15 cm			20 cm		
	exoskeleton stiffness	0 Nm rad^−1^	76 Nm rad^−1^	91 Nm rad^−1^	0 Nm rad^−1^	76 Nm rad^−1^	91 Nm rad^−1^
net ankle work (J kg^−1^)	steady-state	−0.40 ± 0.30	−0.24 ± 0.17	−0.19 ± 0.20	−0.18 ± 0.23	−0.32 ± 0.16	−0.25 ± 0.17
	perturbation^a^	−1.04 ± 0.50	−0.70 ± 0.30	−0.61 ± 0.33 *	−0.64 ± 0.37	−0.76 ± 0.44	−0.79 ± 0.49
positive ankle work (J kg^−1^)	steady-state	0.79 ± 0.20	0.68 ± 0.22	0.72 ± 0.24	0.78 ± 0.21	0.60 ± 0.21	0.67 ± 0.13
	perturbation	0.72 ± 0.24	0.66 ± 0.39	0.70 ± 0.38	0.86 ± 0.37	0.57 ± 0.33	0.63 ± 0.26
negative ankle work (J kg^−1^)	steady-state	1.19 ± 0.44	0.92 ± 0.30	0.92 ± 0.23	0.96 ± 0.42	0.92 ± 0.28	0.92 ± 0.25
	perturbation^a^	1.76 ± 0.49	1.36 ± 0.29 *	1.31 ± 0.55 *	1.49 ± 0.60	1.33 ± 0.62	1.42 ± 0.57
net knee work (J kg^−1^)	steady-state	−0.33 ± 0.16	−0.28 ± 0.10	−0.25 ± 0.17	−0.23 ± 0.18	−0.28 ± 0.14	−0.22 ± 0.11
	perturbation^a^	−0.51 ± 0.29	−0.41 ± 0.23	−0.43 ± 0.29	−0.43 ± 0.26	−0.79 ± 0.33 *	−0.64 ± 0.37
positive knee work (J kg^−1^)	steady-state	0.34 ± 0.16	0.32 ± 0.16	0.35 ± 0.25	0.36 ± 0.24	0.32 ± 0.22	0.35 ± 0.19
	perturbation	0.23 ± 0.19	0.36 ± 0.28	0.39 ± 0.37	0.35 ± 0.29	0.44 ± 0.22	0.37 ± 0.28
negative knee work (J kg^−1^)	steady-state	0.67 ± 0.29	0.92 ± 0.30	0.60 ± 0.39	0.59 ± 0.34	0.60 ± 0.30	0.57 ± 0.25
	perturbation^a^	0.74 ± 0.36	0.77 ± 0.34	0.82 ± 0.54	0.79 ± 0.32	1.24 ± 0.40^b^*	1.01 ± 0.47
net hip work (J kg^−1^)	steady-state	−0.04 ± 0.04	−0.02 ± 0.04	−0.02 ± 0.04	−0.05 ± 0.06	−0.02 ± 0.05	−0.02 ± 0.04
	perturbation^a^	−0.16 ± 0.23	−0.15 ± 0.20	−0.08 ± 0.28	−0.14 ± 0.26	−0.10 ± 0.23	−0.05 ± 0.30
positive hip work (J kg^−1^)	steady-state	0.11 ± 0.04	0.12 ± 0.07	0.11 ± 0.07	0.11 ± 0.06	0.11 ± 0.07	0.11 ± 0.07
	perturbation^a^	0.27 ± 0.24	0.19 ± 0.11	0.25 ± 0.22	0.33 ± 0.25	0.25 ± 0.18	0.38 ± 0.21
negative hip work (J kg^−1^)	steady-state	0.15 ± 0.07	0.92 ± 0.30	0.12 ± 0.11	0.16 ± 0.11	0.13 ± 0.09	0.13 ± 0.08
	perturbation^a^	0.42 ± 0.36	0.34 ± 0.28	0.33 ± 0.26	0.47 ± 0.30	0.35 ± 0.19	−0.43 ± 0.24

^a^Denotes a main effect of the perturbation (*p* < 0.05).

^b^Denotes a main effect of exoskeleton assistance during either the 15 cm or 20 cm perturbation (*p* < 0.05). Significant differences according to the Tukey *post hoc* are denoted by * (*p* < 0.05).

### 3.2. The effect of the perturbation

During the perturbation both with and without the exoskeleton, participants hopped with lower duty factors, due to 25 ± 29 ms longer ground contact and 97 ± 23 ms longer aerial time when compared to steady-state hopping (table 2, all: *p* < 0.001). Participants also hopped to a higher height in the subsequent hop immediately following the perturbation, compared to steady-state hopping (figure 2, *p* < 0.001). Knee and hip angles were on average 5.9 ± 2.9° (*p* = 0.001) and 5.7 ± 2.6° (*p* < 0.001) more flexed during the perturbation, respectively, compared to steady-state hopping (electronic supplementary material, table S1 and figure S1; figure 3). Peak biological ankle dorsiflexion moments were larger during the perturbation, compared to steady-state hopping (*p* < 0.001); however, peak biological ankle plantarflexion moments were not (electronic supplementary material, figure S2; figure 4) . Peak knee extension (positive) and knee flexion (negative) moments were larger during the perturbation, compared to steady-state hopping (electronic supplementary material, table S2, both: *p ≤* 0.042). Similarly, peak hip extension (positive) and hip flexion (negative) moments were larger during the perturbation compared to steady-state hopping (both: *p* = 0.001). Combined, this led to a decrease in total lower limb work during the perturbation (electronic supplementary material, table S3, *p <* 0.001) due to increases in negative work done by individual joints of the lower limb. At the whole-body level, more negative COM work was done during the perturbation, compared to steady-state hopping (electronic supplementary material, table S3, *p =* 0.03).

**Table 2.** Duty factor, ground contact and aerial times during steady-state and perturbation hops with and without exoskeleton assistance. Duty factor, aerial time and ground contact time for steady-state hopping and the perturbation (15 and 20 cm) at 0, 76 and 91 Nm rad^−1^ exoskeleton assistance. Values are reported as mean ± s.d.

	perturbation height	15 cm			20 cm		
	exoskeleton stiffness	0 Nm rad^−1^	76 Nm rad^−1^	91 Nm rad^−1^	0 Nm rad^−1^	76 Nm rad^−1^	91 Nm rad^−1^
duty factor	steady-state	0.59 ± 0.1	0.60 ± 0.08	0.62 ± 0.08	0.60 ± 0.1	0.64 ± 0.07	0.60 ± 0.09
	perturbation^a^	0.51 ± 0.16	0.50 ± 0.12	0.47 ± 0.07	0.52 ± 0.09	0.52 ± 0.1	0.56 ± 0.17
aerial time (s)	steady-state	0.18 ± 0.05	0.17 ± 0.04	0.17 ± 0.04	0.18 ± 0.04	0.15 ± 0.04	0.017 ± 0.04
	perturbation^a^	0.26 ± 0.08	0.27 ± 0.07	0.28 ± 0.05	0.28 ± 0.06	0.27 ± 0.06	0.25 ± 0.1
ground contact time (s)	steady-state	0.27 ± 0.05	0.27 ± 0.03	0.27 ± 0.04	0.27 ± 0.05	0.28 ± 0.04	0.27 ± 0.05
	perturbation^a^	0.30 ± 0.1	0.29 ± 0.09	0.25 ± 0.06^b^*	0.32 ± 0.12	0.30 ± 0.07	0.33 ± 0.11

^a^Denotes a main effect of exoskeleton assistance during either the 15 cm or 20 cm perturbation (*p* < 0.05).

^b^Denotes a main effect of the perturbation (*p* < 0.05). Significant differences according to the Tukey *post hoc* are denoted by * (*p* < 0.05).

## 4. Discussion

In this study, we explored how elastic exoskeletons modulate lower limb joint mechanics in response to rapid unexpected perturbations. We hypothesized that negotiating a vertical perturbation with passive ankle assistance would reduce stability during an unexpected perturbation given that spring-based devices cannot generate nor dissipate energy. Our results highlight that only the 76 Nm rad^−1^ assistance condition at the greatest (20 cm) perturbation height resulted in instability, as indicated by our proxy of stability. In this case, participants were able to regain stability within one hop cycle, suggesting that perturbation recovery is rapid. Furthermore, we report that in response to the perturbation, there was a distal to proximal redistribution of joint work such that the knee takes on an increased energy dissipation role with exoskeleton use. Together, these results highlight that to successfully recover from an unexpected perturbation with ankle exoskeleton assistance, humans use a combination of mechanisms at both the level of the joint and whole-body to maintain stability.

Our results demonstrate that the proportion of negative work done by lower limb joints is altered with the energetic demands of the task (exoskeleton stiffness and perturbation height) (figure 4). Humans adopted a strategy whereby they used the more proximal knee rather than the ankle to dissipate energy during the perturbation. Dick *et al.* [11] conducted similar perturbation experiments without exoskeleton assistance and found that humans adjust lower limb perturbation responses based on the magnitude of the perturbation. Specifically, net energy absorbed at the distal ankle joint increased during small perturbations (5–10 cm), to enable participants to maintain upright rhythmic hopping [11]. However, with increasing perturbation height (20 cm), the proximal joints (knee and hip) performed increasing amounts of negative work to aid in energy absorption [11]. Consistent with this, our results (figure 4*a*) confirm that without exoskeleton assistance, there is a distal to proximal joint work redistribution that occurred during perturbed hopping (15–20 cm). However, in the presence of exoskeleton assistance, the magnitude of this distal to proximal shift increases during the perturbation (15 and 20 cm) — suggesting that perturbations without exoskeletons are modulated primarily by the ankle joint while perturbations with passive ankle exoskeletons are modulated by the knee joint (figure 4*b,c*). Surprisingly, we did not find systematic changes to the redistribution of joint work (i.e. increased knee negative work) with increases in exoskeleton stiffness. Future studies incorporating a wider range of exoskeleton stiffnesses may provide further insights into the relationship between joint-specific energetic requirements and varied exoskeleton assistance.

This redistribution of joint work suggests a proximo-distal gradient in neuromuscular control, which may be related to morphological differences between lower limb muscle-tendon units [27]. Researchers have investigated how bipedal birds modify joint- and whole-body level mechanics [28] and neuromuscular control [27] when encountering an unexpected drop in ground height, camouflaged using tissue paper to avoid visual cues. They demonstrate that birds use a posture-dependant mechanism based on knee extension during initial stance of the perturbed step, whereby the ankle and MTP switch between damper (knee extended) or spring-like behaviour (knee flexed) [27,29]. In terms of neuromuscular control, birds use a feedforward strategy at the hip and knee, while the ankle and MTP rely on feedback control to maintain stability during the perturbation task [27,29]. This enables limb cycling to remain constant, independent of the type of terrain [27,29]. During unanticipated bumps to human walking, perturbation recovery responses were mediated by foot contact, with a higher reliance on the plantarflexor muscles when the bump is encountered with contact of the forefoot and of the quadriceps muscles during rearfoot contact [7]. Further, when humans encounter an increase in surface stiffness (an alteration to stiffness in series to the leg), they respond with a decrease in leg stiffness. This was driven by participants decreasing their ankle stiffness and landing with more flexed knees on stiffer surfaces [30]. In this present study, we observed a proximo-distal gradient in mechanical work with exoskeleton assistance (the alteration to stiffness parallel to the leg), which suggests a similar pattern of altered neuromuscular control. However, it remains unknown if this proximo-distal shift in neuromuscular control is a result of feedforward or feedback control. Users of passive ankle exoskeletons may use a control strategy that minimizes musculoskeletal stresses in the lower limb via shifting absorption from the ankle plantarflexors to the larger more proximal muscles that cross the knee and the hip. Future work using, for example, a combined exoskeleton and constrained knee perturbation task may provide further insights into this potential safety mechanism.

Our results show that despite the inability of elastic exoskeletons to directly dissipate mechanical energy, humans can still effectively dissipate the additional energy of a perturbation, regain stability and recover from a rapid unexpected vertical perturbation. This suggests that passive ankle exoskeletons, which have already shown energetic benefits during steady-state walking [1], may also be suitable for real-world locomotion where humans continuously encounter unexpected disruptions to natural gait due to curbs, holes and bumps within their environment. Future investigations regarding how the perturbation influences muscle-level sensorimotor feedback due to exoskeleton mediated changes [17] in biological ankle stiffness and ankle angle are needed.

There are limitations to the experimental protocol and joint-level analysis techniques implemented in this study. First, we used inverse dynamics to estimate joint moments. This method is limited when co-contraction of antagonist muscles is present. In this case, estimated joint work from inverse dynamics may underestimate (approx. 7%) muscle-tendon positive work [31]. Second, variability in hop height data was used as a proxy for stability. This approach is similar to work conducted by Graboski & Herr who used the mean distance of the centre of pressure between consecutive hops as a proxy of balance during hopping in a full-leg passive exoskeleton [32]. However, other variables such as the number of hops to return to a given energetic threshold (i.e. net zero lower limb work) may yield more direct information on the nature of stability during a perturbation with and without device assistance. Third, the passive device used in this study was of a soft design. Like other soft designs, the shank-device interface led to a reduction in effective device stiffness via either component migration or soft tissue deformation [32]. As a result, exoskeleton moments were smaller than expected. This reduction in exoskeleton moment is an inherent limitation of soft exoskeleton designs and experimental protocols. Fourth, during experimentation, participants were not instructed to reach a target height and were not paced with a metronome. As a result, total hop energy was not controlled between conditions. However, we report no effect of exoskeleton stiffness on hop height or duty factor during steady-state hopping, suggesting that total hop energy between conditions, while not controlled, did not vary between conditions. Finally, we refer to the task as an unexpected perturbation, as participants were blinded to the perturbation timing during the hopping experiments. However, there may have been minimal auditory cues due to the sliding of the wooden platforms along the force plates, confounding our claim that perturbations were truly ‘unexpected’. Using a similar experimental design, Dick *et al.* found limited learning effects due to cues that may have altered the person's response to the perturbation [11].

In this study, we investigated how passive elastic ankle exoskeletons influence the mechanical energetics of the lower limb joints and whole-body during unexpected vertical perturbations to human hopping. When humans encounter rapid, unexpected perturbations in ground height while wearing passive ankle exoskeletons they use a combination of two energy dissipation strategies: (i) hopping higher after the perturbation and (ii) increasing the reliance on more proximal lower limb joints for energy absorption. These results suggest that even though elastic exoskeletons cannot directly dissipate energy, they do not disrupt the perturbation response and humans can recover steady vertical hopping within one cycle. When combined with the known potential for passive ankle exoskeletons to improve economy during steady-state walking [1], our results suggest that similar devices may be suitable for real-world locomotion where humans continuously navigate complex environments and unpredictable terrain. Future work to explore the *in vivo* muscle dynamics and neural control patterns that underpin these joint-level responses will provide insights into the neuromotor strategies used to recover from unexpected perturbations with device assistance.

## Data Availability

Participant average kinematic and kinetic data (*N*=10) for each perturbation height (15 and 20 cm) and exoskeleton stiffness (0, 76 and 91 Nm rad-1) have been included as electronic supplementary material [33].
